# Alveolar Epithelial Cell Dysfunction in Acute Respiratory Distress Syndrome: Mechanistic Insights and Targeted Interventions

**DOI:** 10.3390/biomedicines13092299

**Published:** 2025-09-19

**Authors:** Jing Wang, Jie Chao

**Affiliations:** 1 Department of Physiology, School of Medicine, Southeast University, Nanjing 210009, China; wangjing2016@seu.edu.cn; 2 Jiangsu Provincial Key Laboratory of Critical Care Medicine, Zhongda Hospital, School of Medicine, Southeast University, Nanjing 210009, China

**Keywords:** acute respiratory distress syndrome (ARDS), alveolar epithelial cell (AEC), epigenetic regulation, metabolic reprogramming, nanoparticle delivery, precision medicine

## Abstract

Acute respiratory distress syndrome (ARDS) is a life-threatening condition with high mortality. A central driver in its pathogenesis is alveolar epithelial cell (AEC) dysfunction, which leads to disruption of the epithelial barrier, impaired fluid clearance, and dysregulated inflammatory responses. This review summarizes the key mechanisms underlying AEC injury, including programmed cell death (apoptosis, pyroptosis, necroptosis, ferroptosis), oxidative stress, mitochondrial dysfunction, epigenetic reprogramming (DNA methylation, histone modifications), metabolic rewiring (succinate accumulation), and spatiotemporal heterogeneity revealed by single-cell sequencing and spatial transcriptomics. Multicellular crosstalk involving epithelial–immune–endothelial networks and the gut-lung axis further shapes disease progression. Building on these mechanistic foundations, we evaluate emerging AEC-targeted interventions such as pharmacologic agents (antioxidants, anti-inflammatories), biologics (mesenchymal stem cells and engineered exosomes), and gene-based approaches (adeno-associated virus and CRISPR-Cas9 systems delivered via smart nanocarriers). Complementary strategies include microbiome modulation through probiotics, short-chain fatty acids, or fecal microbiota transplantation, and biomarker-guided precision medicine (e.g., sRAGE, exosomal miRNAs) to enable promise individualized regimens. We also discuss translational hurdles, including nanotoxicity, mesenchymal stem cell (MSC) heterogeneity, and gene-editing safety, and highlight future opportunities involving AI-driven multi-omics, lung-on-chip platforms, and epithelium-centered regenerative therapies. By integrating mechanistic insights with innovative therapeutic strategies, this review aims to outline a roadmap toward epithelium-targeted, precision-guided therapies for ARDS.

## 1. Introduction

Acute respiratory distress syndrome (ARDS) represents a major challenge in critical care medicine, characterized by high morbidity, mortality, and healthcare burden. Its early features primarily include alveolar epithelial cell (AEC) injury, alveolar-capillary barrier dysfunction, and the activation of an acute inflammatory response [1,2]. These pathological changes lead to pulmonary edema, hypoxemia, and respiratory failure. Mechanisms such as apoptosis and autophagy play critical roles in ARDS progression, not only damaging the lungs but also promoting multi-organ dysfunction, which further increases mortality [3,4,5]. Even among survivors, long-term complications are common, including pulmonary fibrosis (PF), impaired lung function, chronic fatigue, muscle weakness and cognitive impairment. These sequelae greatly reduce patients’ quality of life and impose a substantial burden on families and healthcare systems [3,6]. A deeper understanding of ARDS pathogenesis and the development of targeted therapies are expected to improve clinical outcomes and enhance patients’ quality of life. Progress in this area may also alleviate the broader economic and psychosocial burdens.

AECs, as key components of the alveolar-capillary barrier, play a vital role in the pathophysiology of ARDS. The functions of the alveolar-capillary barrier include maintaining barrier integrity, regulating fluid balance, and participating in the immune response [3,7]. There are two primary types of AECs including alveolar type I epithelial cells (AT1 cells) and alveolar type II epithelial cells (AT2 cells). AT1 cells account for 95% of the alveolar surface area with a flat morphology that covers most of the alveolar surface. Their main function is to form a thin and wide barrier to facilitate gas exchange. In contrast, AT2 cells are cuboidal in shape, constitute about 5% of the alveolar surface area, and are interspersed among AT1 cells. AT2 cells perform multiple essential functions, including the secretion of pulmonary surfactant, regulation of fluid balance, and serving as progenitor cells for alveolar repair [8,9]. The advent of advanced technologies, such as single-cell RNA sequencing, spatial transcriptomics, and metabolomics, has significantly advanced our understanding of AEC heterogeneity, plasticity, and dynamic responses during ARDS. These technologies help elucidate the molecular mechanisms driving epithelial dysfunction and identify novel therapeutic targets.

In this review, we comprehensively examine the mechanistic underpinnings of AEC dysfunction in ARDS, including oxidative stress, programmed cell death, epigenetic reprogramming, and metabolic alterations. We further highlight the spatiotemporal dynamics of AECs and their crosstalk with immune and endothelial cells. Finally, we summarize emerging epithelial-targeted interventions, from pharmacologic agents and biologics to gene-based therapies, and discuss their translational potential and challenges. This review aims to bridge fundamental insights with innovative therapies to guide future precision medicine approaches in ARDS.

## 2. Alterations of Epithelial Cell Function During Ards

**Figure 1:**
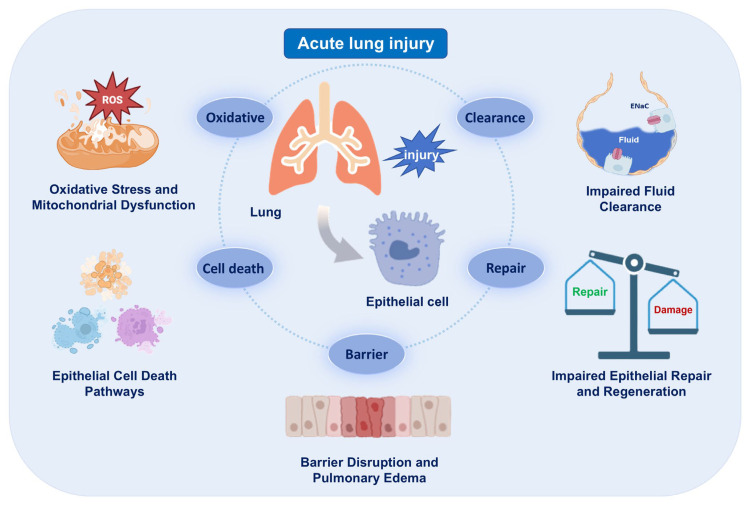
Epithelial injury and its consequences in acute respiratory distress syndrome (ARDS). Schematic summarizing major epithelial phenotypes (cell death, mitochondrial dysfunction, impaired fluid clearance) and their impact on barrier integrity. The images illustrated in the figures were adapted from https://app.biorender.com.

During the pathogenesis of ARDS, AECs serve as the main targets of injury. Their structural and functional disruption compromises the integrity of the alveolar–capillary barrier, leading to pulmonary edema, impaired gas exchange and the amplification of inflammatory cascades. Damaged AECs undergo various forms of programmed cell death, oxidative damage and mitochondrial dysfunction, and exhibit impaired fluid clearance and regenerative capacity. This chapter provides a systematic overview of key pathological processes involving AECs, highlighting the contribution of epithelial dysfunction to ARDS progression (Figure 1).

### 2.1. Barrier Disruption and Pulmonary Edema

Epithelial barrier disruption in ARDS can lead to alveolar edema, inflammatory cell infiltration and impaired gas exchange [10,11]. During the acute phase, inflammatory cells such as neutrophils and macrophages accumulate and release proteases, which cleave junctional proteins (such as E-cadherin) and degrades the extracellular matrix (ECM) [12,13]. The release of mediators such as tumor necrosis factor-α (TNF-α) and interleukin-1β (IL-1β) triggers the rearrangement of actin cytoskeleton in AECs, further increasing permeability [14,15]. As a result, protein-rich fluid leaks into the alveolar space, resulting in pulmonary edema. ARDS also induces oxidative stress that contributes to lipid peroxidation and membrane damage in AECs [16,17]. The detailed mechanisms of oxidative injury and mitochondrial dysfunction are discussed in Section 2.3. In addition, evidence indicates that impaired alveolar fluid clearance further exacerbates pulmonary edema, and the detailed mechanisms of fluid clearance dysfunction are discussed in Section 2.4.

### 2.2. Cell Death Pathways

In ARDS, loss of AECs via programmed cell death not only compromises barrier integrity but also drives inflammation and edema. Apoptosis occurs through intrinsic and extrinsic pathways. Cellular stress (e.g., DNA damage) activates the intrinsic pathway via Bax/Bak-mediated mitochondrial outer membrane permeabilization (MOMP), releasing cytochrome c to form the APAF-1 apoptosome, which activates caspase-9 to cleave and activate executioner caspases-3/7. Death receptors (e.g., Fas/TNFR) initiate the extrinsic pathway by assembling the DISC complex with Fas-associated death domain protein (FADD) and procaspase-8, activating caspase-8. These pathways cause DNA fragmentation, cell shrinkage, and apoptotic body formation. In AECs, excessive apoptosis induces cell detachment, disrupting intercellular junctions and compromising barrier function in ARDS [18,19,20,21].

Pyroptosis is a programmed cell death pathway triggered by inflammasome assembly such as NLRP3. The classical pathway depends on caspase-1 activation, while the non-classical pathway is mediated by caspase-4/5 (human) or caspase-11 (mouse). Activated caspases cleave gasdermin D (GSDMD), and the released GSDMD-N-terminal domain oligomerization forms pores in the plasma membrane, leading to osmotic lysis of cells. This process also promotes the maturation and release of IL-1β and IL-18, thereby amplifying the inflammatory cascade [22,23,24].

Necroptosis of AECs is mediated by the RIPK1-RIPK3-MLKL signaling axis. When AECs are stimulated by pathogen-associated molecular patterns (PAMPs) or damage-associated molecular patterns (DAMPs), RIPK3 activates and phosphorylates mixed lineage kinase domain-like protein (MLKL). Phosphorylated MLKL oligomerizes and translocates to the plasma membrane, forming permeable pores. This causes the loss of cell membrane integrity and release of intracellular contents, directly damaging the alveolar-capillary barrier and exacerbating ARDS pathology [25,26,27].

Additionally, ferroptosis is an iron-dependent form of cell death driven by lipid peroxidation and GPX4 inactivation. It contributes to epithelial damage under oxidative stress and overlaps with mitochondrial dysfunction in ARDS.

### 2.3. Oxidative Stress and Mitochondrial Dysfunction

In ARDS, mitochondrial dysfunction triggers pathological reactive oxygen species (ROS) overproduction in AECs, subverting antioxidant defenses [28]. The resulting oxidative storm impairs cellular function by disrupting membrane integrity via lipid peroxidation, inactivating critical enzymes via protein carbonylation, and inducing bioenergetic failure via mtDNA damage. These alterations synergize with ROS-mediated Nrf2 suppression and *PINK1*/Parkin mitophagy impairment to sustain self-amplifying oxidative stress [21,29,30]. ROS can also alter intracellular signaling proteins and transcription factors, impair normal cell metabolism and promote apoptosis [31,32]. Meanwhile, decreased activities of key antioxidant enzymes further reduce ROS clearance, aggravating oxidative damage [33]. Together, these mechanisms lead to loss of epithelial barrier integrity, trigger programmed cell death and critically drive ARDS progression. For the functional consequences of ROS on epithelial ion transport and alveolar fluid clearance, see Section 2.4.

### 2.4. Impaired Fluid Clearance

AECs maintain alveolar dryness for efficient gas exchange by regulating ion and water transport through epithelial sodium channels (ENaC), Na^+^/K^+^-ATPase and aquaporin 5 (*AQP5*). Impaired fluid clearance also delays epithelial repair and promotes fibroblast activation, thereby increasing the risk of PF [34,35]. In ARDS, a combination of factors leads to impaired fluid clearance in AECs. Pro-inflammatory factors can lead to the degradation of tight junctions, increased barrier permeability, and inhibition of ENaC expression and activity [36,37,38,39]. Excessive ROS produced by macrophages, neutrophils and damaged AECs inactivate ENaC and reduce Na^+^/K^+^-ATPase activity [40,41]. TNF-α inhibits *AQP5* transcription through Nuclear Factor Kappa-Light-Chain-Enhancer of Activated B Cells (NF-κB), and High Mobility Group Box 1 (HMGB1) down-regulates *AQP5* transcription through Toll-like receptor 4 (TLR4) [42,43].

In addition, AT2 cells possess the ability to proliferate and differentiate into AT1 cells, but this ability is inhibited in ARDS due to oxidative stress and inflammatory factors. This inhibition leads to reduced secretion of surface-active substances, including surfactant proteins A (SP-A) and surfactant proteins D (SP-D), leading to increased alveolar collapse and fluid retention [44,45], in line with the functional roles of AT2 cells as described in the Introduction (Section 1). Persistent obstacles to fluid clearance lead to increased fibroblast activation and collagen deposition, further damaging the alveolar structure [46].

### 2.5. Impaired Epithelial Repair and Regeneration

During ARDS, the balance between AEC damage and repair influences disease progression and prognosis. An imbalance in this process contributes directly to respiratory failure. Inflammatory factors, such as Transforming Growth Factor-Beta (TGF-β), and oxidative stress could inhibit the proliferation of AT2 cells [47,48]. The dysregulation of the Wnt/β-catenin pathway impairs the differentiation of AT2 cells into AT1 cells, thereby compromising their regenerative capacity [49]. Furthermore, the reduced secretion of SP-A and SP-D by AT2 cells promotes alveolar collapse and weakens immune defense [49]. Injury results in aberrant DNA methylation in the promoter region of repair-related genes, leading to their repressed transcription [50]. Furthermore, enhanced histone deacetylase (HDAC) activity silences pro-repair genes such as *AQP5*. Collectively, these disruptions to AEC repair mechanisms exacerbate the imbalance between cellular damage and repair, thereby driving disease progression [51,52].

## 3. Tools and Approaches Enabling New Insights

This section concisely overviews the key experimental and computational platforms discussed in this review. It focuses on single-cell transcriptomics, spatial omics, and single-cell and spatial metabolomics. These technologies establish the technical foundation for the mechanistic insights detailed in Section 4.

### 3.1. Single-Cell RNA Sequencing (ScRNA-Seq) for Resolving Cell States and Trajectories

scRNA-seq measures gene expression in individual cells. It is a powerful tool for uncovering cellular heterogeneity, rare transitional states like *Krt8+* alveolar differentiation intermediates, and injury-related differentiation trajectories. Standard analytical workflows typically involve sequential steps including quality control, normalization, dimensionality reduction, clustering, and trajectory inference. Researchers should consider key limitations when interpreting scRNA-seq data, such as capture bias, transcript dropout, and the loss of spatial context [53,54].

### 3.2. Spatial Transcriptomics and Multiplexed Proteomics Recovering Spatial Context

Spatial methodologies bridge the gap left by scRNA-seq by providing crucial spatial context. Several techniques map molecular data directly onto tissue structures. These include in situ hybridization panels, untargeted spatial transcriptomics, and imaging mass cytometry, together offering a complementary perspective to single-cell data. These techniques help localize distinct cell states to specific anatomical niches and reveal spatial patterns in cytokine or metabolite distribution. Each technology involves a balance between resolution, molecular depth, and throughput. Integration with single-cell data from dissociated cells often produces the most comprehensive biological insights [55,56].

### 3.3. Single-Cell and Spatial Metabolomics with Multi-Omic Integration

Emerging approaches in single-cell metabolic inference and spatial metabolomics enable direct correlations between transcriptional states and metabolic functions. Examples include detecting succinate accumulation or inferring succinate dehydrogenase (SDH) complex dysfunction [57,58]. Multi-omic integration of transcriptomic, epigenomic, and metabolomic data enables more robust testing of mechanistic hypotheses. Current technical challenges include limited sensitivity in metabolite detection at cellular resolution and computational challenges in cross-modal data integration. To validate these findings, orthogonal approaches such as targeted biochemistry or perturbation experiments are essential [59,60].

Section 4 presents specific applications and key findings derived from these platforms, including work on succinate dehydrogenase A (*SDHA*)/succinate and alveolar differentiation intermediates. The reader is directed to the methodological introductions above for technical context.

## 4. Key Molecular Mechanisms and Cellular Crosstalk in ARDS

In ARDS, AECs are governed by an intricate interplay of intracellular signaling cascades, epigenetic reprogramming, and metabolic adaptations. Based on the single-cell and spatial multi-omics technologies introduced in Chapter 3, the following sections detail the mechanistic insights these tools have enabled (Table 1). In this chapter, we first dissect the activation dynamics and crosstalk among pro-inflammatory pathways, then investigate how epigenetic modifiers such as DNA methylation shifts, histone modifications and non-coding RNAs, reshape transcriptional landscapes. Subsequent sections explore metabolic rewiring to accelerated glycolysis and fatty acid oxidation (FAO), and finally analyze the bidirectional crosstalk between epithelium and immune cells that orchestrates tissue injury and repair processes.

### 4.1. Activation of Inflammatory Signaling Pathways

In ARDS, a complex network exists within AECs where multiple signaling pathways cross-regulate and amplify inflammatory responses, ultimately leading to cellular dysfunction and apoptosis.

First, the binding of TNF-α or IL-1β rapidly activates the IκB kinase (IKK) complex, enabling nuclear translocation of NF-κB and driving transcription of pro-inflammatory and pro-apoptotic genes such as *TNF* and *IL-6* [61]. Second, the c-Jun N-terminal kinase (JNK), a classical mitogen-activated protein kinase (MAPK) family member, is persistently activated in the inflammatory milieu. Activated JNK directly phosphorylates Bcl-2 family proteins, triggering mitochondria-mediated apoptosis, while p38 MAPK contributes to lung injury by regulating inflammatory gene transcription and inducing cell cycle arrest [62]. Finally, IL-6 family cytokines engage their receptors to activate the signal-transducing GP130 subunit and Janus kinase (JAK) tyrosine kinases, leading to Signal Transducer and Activator of Transcription 3 (STAT3) phosphorylation, dimerization, and nuclear accumulation. STAT3 then enhances pro-inflammatory gene transcription, amplifying the inflammatory cascade [63,64]. Notably, NF-κB, MAPK, and JAK/STAT3 pathways engage in cross-regulation. For example, NF-κB can up-regulate JAK/STAT3 components, while STAT3 modulates NF-κB activity, collectively driving ARDS initiation and progression [65,66].

Overall, these synergistic pro-inflammatory signaling pathways establish an efficiently amplifiable and tightly regulated inflammatory network, representing a pivotal molecular mechanism in ARDS pathogenesis.

### 4.2. Epigenetic Regulation

**Figure 2:**
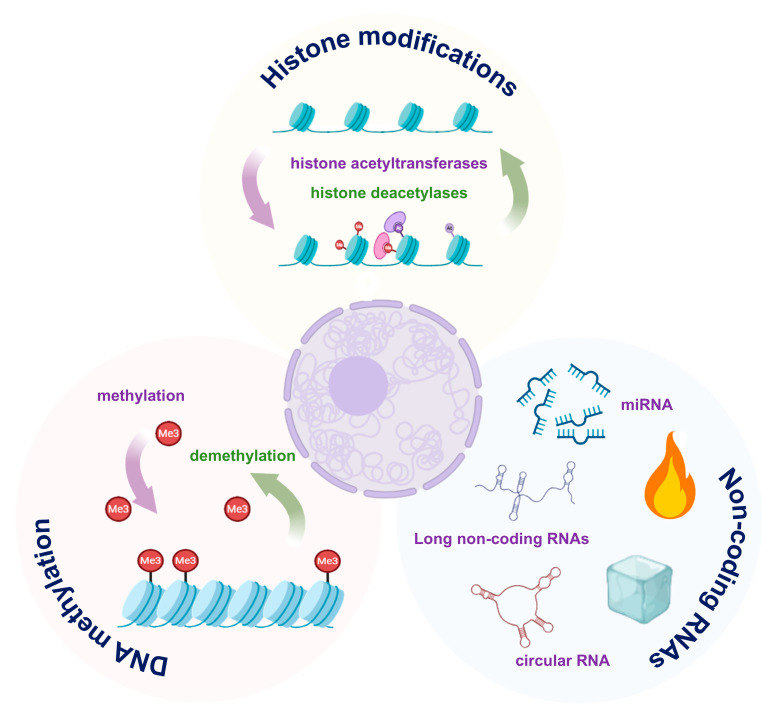
Major epigenetic mechanisms in alveolar epithelial cell (AEC) dysfunction. DNA methylation, histone modifications and non-coding RNAs modulate pro-inflammatory and pro-repair gene programs. The images illustrated in the figures were adapted from https://app.biorender.com.

In ARDS, epigenetic regulation (Figure 2), including DNA methylation, histone modifications, and non-coding RNAs, emerges as a key mechanism driving AEC injury, by modulating gene expression without altering the DNA sequence [67,68].

**Table 1:** Summary of Key Molecular Mechanisms in AEC Dysfunction during ARDS.

Mechanism	Affected Aec Process	Key Molecular Players/Pathway	References
Pro-inflammatory signaling	Cytokine production, apoptosis, barrier dysfunction	NF-κB, JNK/p38 MAPK, JAK/STAT3, TNF-α, IL-1β, IL-6	[52,61,62,63,64,65]
Programmed cell death	Cell death, release of DAMPs, propagation of inflammation	Caspase-8, MLKL (Necroptosis), GSDMD (Pyroptosis)	[66,67]
Epigenetic reprogramming	Transcriptional silencing of repair genes, sustained inflammatory gene expression	DNA methylation (*AQP5*), HATs/H3K27ac, HDACs, H3K4me3, H3K27me3, miR-155, miR146a	[69,70,71]
Metabolic reprogramming	Energy production, succinate signaling, HIF-1α activation	Impaired FAO, SDHA dysfunction, Succinate accumulation, Glycolytic switch, *HIF-1α*	[72,73,74,75]
Epithelial–immune–endothelial crosstalk	Immune cell recruitment, barrier integrity, fibrotic remodeling	CCL2, CXCL1, CXCL8, VEGF, HMGB1, VE-cadherin	[75,76,77,78,79,80,81,82,83]
Gut-Lung Axis	Barrier function, inflammasome activation, systemic inflammation	SCFAs/Butyrate (protective), TMAO (detrimental), NLRP3	[84,85,86,87,88,89,90,91]

AEC, alveolar epithelial cell; ARDS, acute respiratory distress syndrome; *AQP5*, aquaporin-5; CCL2, C-C motif chemokine ligand 2; CXCL1, C-X-C motif chemokine ligand 1; DAMPs, damage-associated molecular patterns; FAO, fatty acid oxidation; GSDMD, gasdermin D; HATs, histone acetyltransferases; HDACs, histone deacetylases; *HIF-1α*, hypoxia-inducible factor 1-alpha; HMGB1, high mobility group box 1; IL, interleukin; JAK/STAT3, Janus kinase/signal transducer and activator of transcription 3; JNK, c-Jun N-terminal kinase; MAPK, mitogen-activated protein kinase; MLKL, mixed lineage kinase domain-like; NF-κB, nuclear factor kappa-light-chain-enhancer of activated B cells; NLRP3, NLR family pyrin domain containing 3; SCFAs, short-chain fatty acids; *SDHA*, succinate dehydrogenase complex flavoprotein subunit A; TMAO, trimethylamine N-oxide; TNF-α, tumor necrosis factor-alpha; VE-cadherin, vascular endothelial cadherin; VEGF, vascular endothelial growth factor.

#### 4.2.1. DNA Methylation

During the progression of ARDS, a portion of the DNA undergoes methylation, resulting in the silencing of repair genes and the activation of pro-inflammatory pathways. Stress-induced hypermethylation of key repair factors has been shown to suppress AT2 cell proliferation and their differentiation into AT1 cells.

Conversely, hypomethylation of inflammatory genes maintains cytokine production and barrier disruption [69,70]. *AQP5* promoter methylation was observed to result in reduced expression of water channel proteins and impaired fluid clearance [71]. Further studies revealed that promoter demethylation of pro-inflammatory genes enhances their transcriptional activity, thereby amplifying the inflammatory response [72].

#### 4.2.2. Histone Modifications

Histone modifications, such as acetylation and methylation, are critical epigenetic regulators of chromatin remodeling and gene expression. They play a key role in the pathogenesis of ARDS. In response to inflammatory stimuli, the recruitment of histone acetyltransferases (HATs) promotes H3K27 acetylation (H3K27ac) at promoters of pro-inflammatory genes. This open chromatin state facilitates NF-κB-driven transcription of cytokines, thereby exacerbating epithelial injury [73]. Conversely, activation of histone deacetylases (HDACs) removes acetyl groups from the promoters of anti-inflammatory and reparative genes (e.g., IL-10, SP-A), leading to their transcriptional silencing.

Beyond acetylation, alterations in histone methylation also contribute to dysregulated inflammation and impaired repair. For instance, persistent inflammatory responses have been linked to aberrant histone methylation marks, including the activating H3K4me3 and the repressive H3K27me3, which are enriched at the promoters of pro-inflammatory genes [74,75]. Moreover, the deposition of the repressive mark H3K27me3 at promoters of epithelial repair genes effectively inhibits AT2 cell proliferation. Notably, inhibition of the H3K27 methyltransferase was shown to reverse this repression, restore reparative gene expression, and promote alveolar regeneration [76,77].

#### 4.2.3. Non-Coding RNAs

Non-coding RNAs regulate inflammation and cell fate. Pro-inflammatory miRNAs, such as *miR-155*, amplify inflammatory responses by inhibiting SOCS1 expression and enhancing JAK-STAT pathway activity [78,79]. In contrast, anti-inflammatory miRNAs, such as *miR-146a*, have been found to target TRAF6/IRAK1 and negatively regulate NF-κB signaling [80]. Long non-coding RNAs (lncRNAs) also affect AEC function through interactions with chromatin [81].

### 4.3. Metabolic Reprogramming

Based on the single-cell and spatial metabolomics tools introduced in Chapter 3, the following summarizes specific findings on epithelial metabolic rewiring in ARDS. Impairment of FAO in AECs has been shown to exacerbate ALI. Conversely, AECs may ameliorate lung inflammation by modulating mitochondrial FAO [82,83]. Recent single-cell transcriptomic analyses in murine acute lung injury (ALI) models have revealed that dysfunction of *SDHA* leads to intracellular succinate accumulation and Hypoxia-Inducible Factor 1 Alpha Subunit (*HIF-1α*) stabilization, driving a metabolic switch of AT2 cells toward glycolysis [84,85]. In a mechanical ventilation induced ALI model, AT2-specific deletion of *SDHA* (sdhaloxp/loxp SPC-CreER mice) recapitulated succinate accumulation and *HIF-1α* activation, and improved barrier integrity with reduced epithelial injury [84]. Accumulated succinate not only alters epithelial metabolism but also acts as a paracrine signal, and its SUCNR1-mediated immunoregulatory effects are discussed in Section 5.4.

Complementing these single-cell insights, spatial metabolomics techniques are beginning to map succinate gradients and other small molecule distributions in injured lung tissue. Although comprehensive imaging studies in classic ALI models such as lipopolysaccharide (LPS) or bleomycin (BLM) remain limited, the existing imaging methods have demonstrated the feasibility of locating small molecules under inflammatory conditions. Collectively, these findings underscore that epithelial metabolic reprogramming not only reflects injury severity but actively shapes the immune microenvironment. Integrating spatial metabolomics with single-cell multi-omics will be essential for dissecting the dynamic metabolite-cell interactions that drive ALI progression and for identifying novel therapeutic targets.

### 4.4. Epithelial Heterogeneity and Plasticity

Following the metabolic changes summarized above, scRNA-seq and spatial transcriptomics provide the cellular resolution needed to define transitional states and repair trajectories. Emerging technologies such as single-cell RNA sequencing (scRNA-seq) and spatial transcriptomics have transformed our understanding of ARDS by shifting from bulk “population” analyses to integrated high-resolution spatiotemporal maps of epithelial diversity and dynamics. They provide real-time insights into both the emergence of distinct AEC subpopulations during injury and repair, and the impact of their spatial context and interactions with neighboring cells and the ECM on disease progression.

Following lung injury, scRNA-seq studies have shown that airway and alveolar progenitors do not fully differentiate into mature AT1 cells but instead converge on a *Krt8*+ “alveolar differentiation intermediate” (ADI) transitional state. Spatial transcriptomics further localizes these ADI cells predominantly at the alveolar-bronchiolar junction, colocalizing with fibroblasts and suggesting their involvement in early fibrotic processes [81,86]. In animal models, activation of Yap/Taz signaling drives expansion of *Krt8*+ cells and accelerates alveolar repair, whereas epithelial-specific deletion of Yap and Taz blocks AT2 differentiation, resulting in persistent collagen deposition, neutrophilic inflammation and impaired lung function [86,87]. Moreover, single-cell trajectory analyses reveal that failed repair correlates with upregulation of pro-fibrotic gene programs and dysregulation of Wnt/β-catenin signaling, suggesting Wnt/β-catenin imbalance as a critical barrier to effective regeneration [86,88,89].

Together, these findings not only deepen mechanistic insights into ARDS pathogenesis but also highlight Yap/Taz and Wnt/β-catenin pathways as promising targets for precision interventions.

### 4.5. Epithelial–Immune–Endothelial Crosstalk

Building on the epithelial heterogeneity and metabolic rewiring discussed above, we next examine how AECs communicate with immune and endothelial cells to orchestrate ARDS pathogenesis. In ARDS, AECs, immune cells and endothelial cells (ECs) form a dynamic multicellular network that governs both inflammation and barrier repair. Recent scRNA-seq and spatial transcriptomics studies have uncovered previously unrecognized heterogeneity among AT2 cells, identifying subpopulations with high expression of pro-inflammatory cytokines and chemokines such as CCL2, CXCL1 and CXCL8. These AT2 subsets actively orchestrate immune cell recruitment and fuel local inflammation [11,87].

Moreover, spatial omics coupled with high parameter imaging has precisely mapped cytokine rich epithelial micro niches (“hotspots”) in situ. For instance, GeoMx Digital Spatial Profiling revealed that AECs exhibit upregulation of cytokines and matrix-remodeling genes, coinciding with dense CD68^+^macrophage infiltration in SARS-CoV-2–associated ARDS [90]. Inflammatory alveolar macrophage-derived microvesicles have been implicated in damaging AECs, thereby promoting pulmonary edema and exacerbating lung injury [91]. Imaging mass cytometry further revealed spatial colocalization of AECs with both CD15+CD11b+ polymorphonuclear neutrophils and CD68+ macrophages, frequently near ECs. Spatial network analyses then revealed that these ECs actively participate in recruiting and activating local immune cells. Together, these findings underscore a critical epithelial–immune–endothelial crosstalk driving early inflammatory and fibrotic remodeling in ARDS [92,93].

Beyond epithelial-immune interactions, ECs play a central role in modulating vascular permeability and immune cell extravasation. Injured AECs release key mediators such as Vascular Endothelial Growth Factor (VEGF), and DAMPs such as HMGB1. These molecules disrupt endothelial junctional proteins such as Vascular Endothelial Cadherin (VE-cadherin), increasing capillary leak. Conversely, activated ECs secrete IL-6 and granulocyte-macrophage colony-stimulating factor (GM-CSF), amplifying leukocyte recruitment and perpetuating epithelial injury [94,95,96].

**Figure 3:**
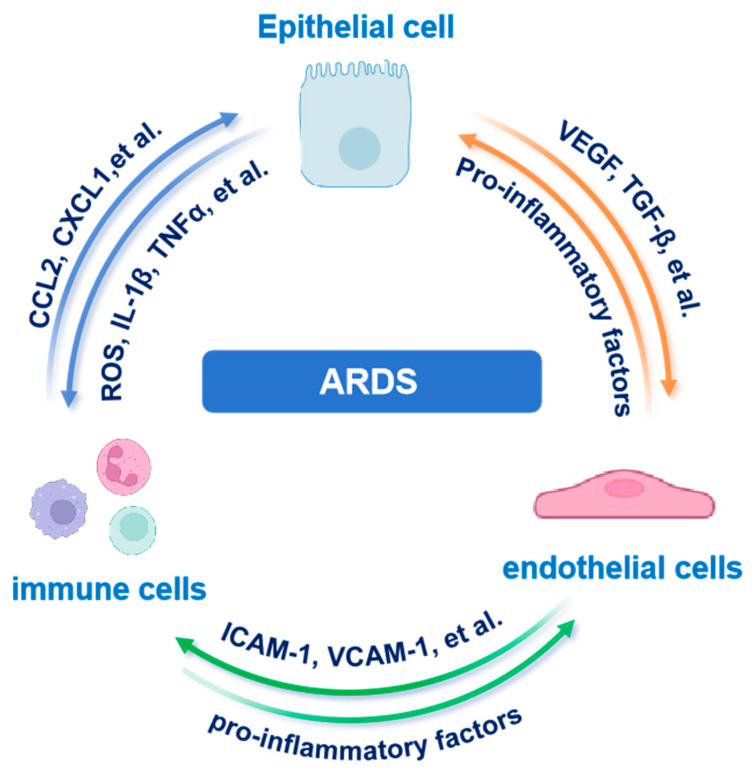
Tripartite epithelial–immune–endothelial crosstalk in ARDS. AEC-derived chemokines recruit immune cells that amplify endothelial leak and further epithelial injury. Abbreviations: AEC, alveolar epithelial cell; ARDS, acute respiratory distress syndrome; CCL2, C-C motif chemokine ligand 2; CXCL1, C-X-C motif chemokine ligand 1; ICAM-1, intercellular adhesion molecule 1; IL-1β, interleukin-1 beta; TGF-β, transforming growth factor beta; TNFα, tumor necrosis factor alpha; VCAM-1, vascular cell adhesion molecule 1; VEGF, vascular endothelial growth factor. The images illustrated in the figures were adapted from https://app.biorender.com.

Collectively, these bidirectional signals establish a self-perpetuating inflammatory loop (Figure 3). Epithelial-derived chemokines initiate immune cell recruitment and activation. Immune-derived mediators, including cytokines and ROS, subsequently disrupt endothelial integrity, potentiating immune infiltration and epithelial injury. Spatially resolved studies define multicellular hubs (AECs, immune cells, ECs) as sites where triadic crosstalk drives fibrotic remodeling and barrier dysfunction in ARDS. Moreover, transplantation of human embryonic stem cell-derived AT2 cells has been shown to alleviate ARDS in mouse models, further supporting the important role of AECs in lung homeostasis and injury resolution [97].

### 4.6. Modulation of Alveolar Epithelium by the Gut-Lung Axis

Beyond local cell–cell interactions, systemic modulators including the gut-lung axis can influence epithelial resilience and repair. The gut-lung axis plays a critical role in maintaining pulmonary homeostasis and alveolar epithelial integrity during ARDS. Dysbiosis of the gut microbiota and increased intestinal permeability facilitate the translocation of microbial products and viable bacteria into the systemic circulation. This process amplifies systemic inflammation, exacerbates AEC injury, and disrupts barrier function [98,99]. Preclinical studies have shown that depletion of beneficial taxa, such as *Akkermansia muciniphila*, worsens alveolar damage, whereas probiotic supplementation restores epithelial integrity, highlighting the distal regulatory role of the gut microbiota on lung epithelium [100,101].

Moreover, microbiota-derived metabolites directly influence AEC function. Short-chain fatty acids (SCFAs), particularly butyrate, enhance mitochondrial activity and upregulate tight junction proteins such as ZO-1, thereby mitigating alveolar edema. In contrast, dysbiosis-associated metabolites, including trimethylamine N-oxide (TMAO), promote NLRP3 inflammasome activation and pyroptosis in AECs [101,102,103]. These findings underscore the therapeutic potential of microbiome-targeted interventions. Strategies such as fecal microbiota transplantation (FMT), SCFA analogs, or engineered probiotics have shown promise in preserving alveolar-capillary barrier function and improving outcomes in preclinical ALI models [104,105].

## 5. Therapeutic Strategies Targeting Epithelial Cells

**Figure 4:**
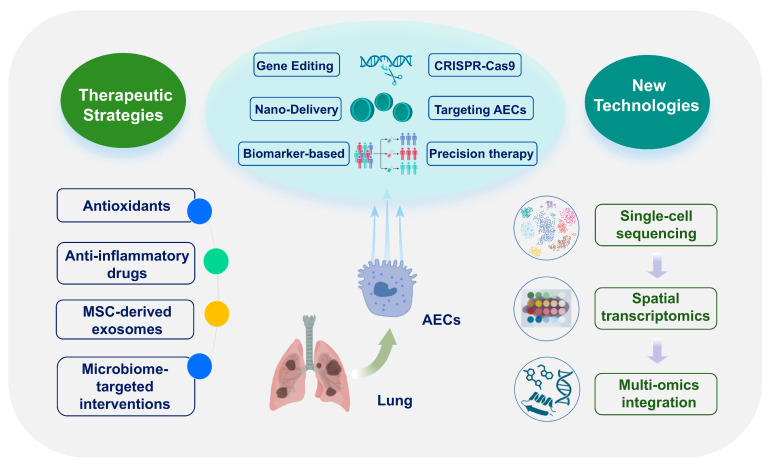
Epithelial-targeted therapeutic strategies. Overview of antioxidants, biologics, cell/gene therapies, nanodelivery, microbiome interventions and biomarker-guided precision approaches. Abbreviations: AEC, alveolar epithelial cell; MSC, mesenchymal stem cell. The images illustrated in the figures were adapted from https://app.biorender.com.

AEC dysfunction is a key driver of alveolar-capillary barrier failure and immune dysregulation in ARDS and represents a critical target for therapeutic intervention. This chapter synthesizes mechanistically targeted strategies to preserve AEC function, including antioxidant and anti-inflammatory agents, cell and gene therapies, advanced delivery systems, microbiome modulation, and biomarker-guided precision medicine (Figure 4 and Table 2).

### 5.1. Antioxidant and Anti-Inflammatory Therapies

#### 5.1.1. Antioxidants

Oxidative stress is a key factor in AEC damage during ARDS, and antioxidant therapy has been shown to alleviate this damage. N-acetylcysteine (NAC), a precursor of glutathione, has been shown to effectively reduce oxidative stress and maintain the integrity of AEC membranes. In the trial registered as NCT04374461 and in several small randomized or open-label studies, NAC was associated with reductions in oxidative-stress biomarkers and, in some reports, modest improvements in oxygenation. However, these small studies did not consistently demonstrate benefit on hard clinical endpoints such as mortality or ventilator-free days. Overall, the data indicate a biological effect of NAC but highlight the need for larger, biomarker-guided trials to determine whether these antioxidant effects translate into clear clinical benefit [106,107]. Similarly, melatonin has shown protective effects in ALI models by activating the nuclear factor erythroid 2-related factor 2 signaling pathway and enhancing the expression of key antioxidant enzymes, thereby reducing alveolar edema and inflammatory responses [108,109]. These findings support the therapeutic potential of antioxidants in limiting epithelial damage mediated by oxidative stress in ALI.

#### 5.1.2. Anti-Inflammatory Drugs

Glucocorticoids remain the cornerstone of anti-inflammatory therapy for ARDS, and they work mainly by inhibiting the NF-κB signaling pathway, thereby reducing the production of key cytokines such as IL-6 and TNF-α [110,111]. However, prolonged glucocorticoid exposure can compromise epithelial repair and immune surveillance, which underscores the need to carefully balance anti-inflammatory benefits against potential delays in lung recovery.

Another strategy targets specific cytokine axes. The IL-1 receptor antagonist anakinra blocks the IL-1β signaling pathway, preventing epithelial pyroptosis and amplification of downstream inflammation. In the randomized trial registered as NCT04339712 in patients with COVID-19–associated ARDS, anakinra was generally well tolerated and was associated with reductions in inflammatory biomarkers and, in some analyses, reduced ventilator dependence; however, results have been inconsistent across studies, suggesting that benefit may be restricted to biomarker-defined subgroups and that prospective, stratified trials are needed [112,113].

Together, these approaches illustrate two complementary modes of anti-inflammatory action including broad-spectrum inhibition by glucocorticoids and targeted cytokine blockade, both of which have differential effects on epithelial integrity and repair.

### 5.2. Cell and Gene Therapies

Cell and gene therapies have emerged as promising approaches to restore AEC function and attenuate lung injury in ARDS. Among them, mesenchymal stem cells (MSCs) have been extensively studied due to their immunomodulatory, anti-apoptotic and regenerative properties. Preclinical studies have demonstrated that both systemic and intratracheal administration of bone marrow or adipose-derived MSCs can reduce alveolar permeability, enhance AEC survival, and promote epithelial repair [114,115,116]. These effects are largely mediated by paracrine signaling, including the release of growth factors, cytokines and extracellular vesicles that influence AEC behavior [117,118].

Gene-based interventions provide an additional layer of targeted control over epithelial pathophysiology. Adeno-associated virus (AAV) vectors have been used to deliver genes that encode anti-inflammatory or barrier-protective molecules directly to AECs, thereby improving alveolar stability [119,120]. Furthermore, CRISPR-Cas9 genome editing offers a powerful platform for correcting gene dysfunctions or modulating key signaling pathways involved in epithelial injury and regeneration [121,122]. However, considerable challenges persist, mirroring the limitations of the pharmacologic approaches noted in Section 5.1. Efficient delivery of therapies remains a major challenge. Transplanted cells often fail to engraft in damaged lung tissue, and viral or non-viral vectors show inconsistent transduction rates. Immunogenicity introduces further risks, including the immune-mediated rejection of allogeneic cells and adverse reactions to gene-editing tools. Safety issues also remain, particularly uncertainties around long-term consequences and off-target effects. Future work should focus on developing integrated strategies, such as biomaterial-assisted delivery, nanocarrier systems, and immune-modulatory regimens, to improve the efficacy and safety of advanced cell and gene therapies.

### 5.3. Exosomes and Nanodelivery Systems

As nature’s inherent nanocarriers, exosomes have attracted significant interest for the treatment of ARDS. This stems from their intrinsic biocompatibility and ability to transport bioactive cargo across the alveolar barrier [123,124]. MSC-derived exosomes demonstrate substantial therapeutic efficacy in diverse ALI models [125]. These exosomes, whether loaded with anti-inflammatory miRNAs (such as *miR-146a-5p*) or engineered to display lung-homing peptides, function by reducing pro-inflammatory cytokines (including TNF-α and IL-1β), enhancing IL-10 secretion, and restoring alveolar barrier integrity [125,126,127].

In parallel, synthetic nano-delivery systems have achieved notable advances. Lipid nanoparticles (LNPs) and polymeric platforms enable precision delivery of therapeutic genes and drugs. Neutrophil membrane-coated poly (lactic-co-glycolic acid) (PLGA) nanoparticles carrying TLR4 siRNA selectively target inflammatory macrophages to suppress NF-κB activation and mitigate LPS-induced lung injury [128]. Mannosylated LNPs delivering *miR-146a* improve survival in hemorrhagic-shock-induced ARDS. And 1,2-dioleoyl-3-trimethylammonium-propane (DOTAP)-modified LNPs encapsulating sPD-L1 mRNA induce lung-restricted immunosuppression in ARDS models [129].

Future research will focus on “intelligent” responsive nanomaterials, which can release therapeutic drugs when the level of reactive oxygen species or proteinase activity increases, thereby achieving precise temporal control of pulmonary inflammation. The synergistic combination of biomimetic exosomes and programmable synthetic nanocarriers provides a transformative approach for individualized treatment of ARDS, but it needs to overcome the obstacle of scaling up production and prove its efficacy in advanced disease models.

### 5.4. Precision Medicine and Biomarkers

In recent years, multi-omics technologies such as scRNA-seq, metabolomics and proteomics have revealed the dynamic changes of biomarkers in blood and bronchoalveolar lavage fluid (BALF). These advances have improved our understanding of ARDS and provided important insights for clinical classification, prognosis, and targeted therapy [130]. The scRNA-seq identified ADIs, which arise during the transformation of AT2 to AT1 cells after lung injury and are associated with epithelial repair. These transitional cells are enriched in fibrotic lungs and exhibit pro-inflammatory and stress-responsive features [86]. Accumulated succinate in injured epithelium not only affects epithelial metabolism but also acts on macrophages through SUCNR1, thereby promoting pro-inflammatory responses, as discussed in Section 4.3 [131]. Soluble receptor for advanced glycation end products (sRAGE) is a biomarker of AT1 cell injury, with elevated levels correlating with disease severity in ARDS [132]. Furthermore, exosomal miRNAs (including *miR-146a*) can serve as potential biomarkers for the intensity of inflammatory response and may help to identify patients who will benefit from anti-inflammatory interventions [126,133]. Finally, scRNA-seq has further revealed epithelial heterogeneity, identified profibrotic AT2 subsets within patient lungs, and facilitated the selection of targeted agents, such as TGF-β inhibitors, for individualized treatment [134,135].

### 5.5. Microbiome Targeted Interventions

ARDS-associated gut–lung axis dysregulation increases intestinal permeability, facilitating microbial products to enter the circulation and worsen alveolar epithelial inflammation. Targeting the microbiome shows preclinical promise. In animal models, specific probiotics such as Lactobacillus rhamnosus GG and Bifidobacterium breve modulate CD4^+^ regulatory T cell (Treg) and T helper 17 cell (Th17) balance, reduce pulmonary inflammation and improve epithelial barrier function [98,136,137]. Short-chain fatty acids (SCFAs), especially butyrate, appear to activate the epithelial free fatty acid receptors GPR41 (also known as FFAR3) and GPR43 (FFAR2), resulting in inhibition of inflammation and facilitating mucus layer repair in these models [138,139]. Fecal microbiota transplantation (FMT) similarly reduces inflammation and mortality in animals. However, its clinical translation encounters challenges in standardization and safety [140,141]. These microbiome interventions show protection in animal ALI models, and high-quality clinical trials are needed to validate their therapeutic potential.

Future research needs to utilize sophisticated tools like humanized mouse models and lung-on-a-chip technology to carefully evaluate probiotic strains and microbial metabolites. The key is finding those that can both stably establish themselves in the gut and actively promote epithelial repair. At the same time, we must develop better ways to deliver these agents directly to the airways and establish standardized methods to consistently measure their effectiveness and safety. These efforts will help move these promising approaches from the lab to the clinic faster.

## 6. Challenges and Future Perspectives

### 6.1. Translational Challenges

The clinical translation of ARDS therapies faces multifaceted technical hurdles. Nano-delivery systems are challenged by low lung-targeting efficiency and systemic toxicity, though surface modifications (such as lung-specific ligands) and nebulized formulations offer promising solutions [142,143,144]. Gene editing precision remains compromised by off-target effects, necessitating high-fidelity Cas9 variants (such as eSpCas9) and AI-optimized guide RNA design to enhance specificity [145,146,147,148]. Concurrently, MSC heterogeneity, driven by donor variability and culture conditions, undermines therapeutic consistency, mandating GMP-compliant cell banking and standardized protocols [149,150,151]. Personalized approaches are hindered by inadequate biomarkers. Integrating multi-omics signatures (such as sRAGE, IL-6, Ang-2, miRNAs) with real-time biosensors and machine learning is critical for dynamic subtyping and treatment optimization [152,153,154].

Safety and ethical complexities further impede translational progress. Cationic lipid-based nanocarriers risk pulmonary inflammation, prompting a shift toward biodegradable polymers or exosomes with improved biocompatibility [155,156,157]. Gene editing vectors (such as AAVs) face immunogenicity barriers, driving exploration of engineered variants and non-viral platforms (such as PiggyBac, mRNA-Cas9) to enable repeated dosing [158,159]. Crucially, CRISPR therapies raise unresolved ethical concerns, including germline contamination and carcinogenic risks, demanding strict somatic-cell editing limits, IRB oversight per Helsinki principles, and long-term registries spanning over 10 years to monitor delayed toxicity [160,161,162,163,164,165,166]. Human-lung-chip platforms provide vital preclinical tools for dose safety validation [167].

### 6.2. Emerging Technologies Driving Mechanistic Insights

#### 6.2.1. Single-Cell and Spatial Omics

The scRNA-seq in bleomycin-injured murine lungs has revealed a population of transitional *Krt8+* alveolar progenitors. These analyses suggest the Hippo-Yap/Taz signaling axis as a therapeutically targetable pathway. Pharmacologic inhibition of Yap/Taz suppresses *Krt8+* cell proliferation, while genetic ablation of *Yap1* severely compromises alveolar regeneration. These complementary approaches indicate that modulating Hippo-Yap/Taz activity may enhance epithelial repair [86,168]. Further leveraging single-cell trajectory analysis, researchers observed an IL-1β–HIF1α–mediated glycolytic shift that traps AT2 cells in a damage-associated transient progenitor (DATP) state. Importantly, interruption of IL-1β or HIF1α signaling reverses this differentiation arrest and promotes AT1 maturation. This highlights cytokine blockade (IL-1β) and metabolic reprogramming (*HIF1α* inhibition) as promising combination therapy for epithelial regeneration [169].

Translating these insights to human pathology, spatial transcriptomic profiling of fibrotic lungs identified localized “fibrotic hotspots”. These niches are characterized by aberrant activation of Notch and TGF-β pathways within *KRT17*^+^ epithelial clusters, co-localized with myofibroblast accumulation and stress-response signatures [170]. This spatial resolution directly informs precision therapeutic strategies, such as aerosolized delivery of TGF-β inhibitors or Notch modulators, designed to selectively disrupt pro-fibrotic signaling within these microenvironments [134,171,172]. Consequently, the integration of single-cell dynamics with spatial architecture offers a powerful framework to identify context-dependent therapeutic targets and advance mechanism-based interventions for ARDS and PF.

#### 6.2.2. Mechanobiology and Microfluidic “Lung-on-Chip” Platforms

Studies in mechanobiology utilizing tunable substrates and cyclic stretch have demonstrated that elevated ECM stiffness and mechanical strain promote Yap/Taz nuclear translocation in AECs. This mechanotransduction pathway critically regulates epithelial barrier integrity and potentiates pro-fibrotic responses [173,174]. To model these dynamics, microfluidic lung-on-chip platforms have been developed. These devices incorporate a stretchable, ECM-coated membrane that mimics the alveolar-capillary interface, subject to controlled fluidic shear stress and cyclic mechanical deformation. This setup enables real-time quantification of key parameters including vascular permeability, transepithelial electrical resistance (TEER), and cell–matrix adhesion kinetics [175,176].

Notably, advanced iterations integrate adjacent microchambers for fibroblast or immune cell co-culture, coupled with embedded biosensors. This design facilitates high-resolution spatiotemporal analysis of mechanobiological crosstalk within a human-relevant microenvironment [177,178]. Collectively, lung-on-chip systems offer a physiologically mimetic platform for deconvoluting epithelial-matrix mechanosignaling and accelerating the discovery of mechano-targeted therapeutics.

#### 6.2.3. AI-Driven Multi-Omics Integration

AI and graph-based computational models are transforming multi-omics integration to decode cell–cell communication networks, significantly accelerating target identification. Techniques spanning random forest-based feature selection to graph neural networks infer key regulatory hubs and pathways obscured in single-omic analyses. For example, integrative metabolomic-proteomic profiling of ALI models identified succinate-*SUCNR1* signaling as a critical immunometabolic driver. Subsequent AI validation prioritized this axis for therapeutic targeting [59,131,179]. By reconstructing disease-relevant networks, these approaches not only pinpoint druggable targets but also predict synergistic drug combinations, accelerating precision medicine design.

### 6.3. Toward Epithelium Centered Precision ARDS Therapy

Current mechanistic insights into AEC injury and repair underpin an epithelium-centered precision paradigm for ARDS. This approach begins with patient stratification through epithelial endotyping. Integrated multi-omics signatures and spatial biomarkers, such as alveolar damage indicators, sRAGE, and exosomal miRNA profiles, are utilized to classify patients into hyper-inflammatory, metabolically dysregulated or fibrotic-prone subgroups [175,180]. Endotype classification provides a mechanistic framework for therapeutic selection. Hyper-inflammatory states may be targeted by IL-1β or NF-κB pathway inhibitors. For metabolically dysregulated endotypes, *SUCNR1* antagonists or *HIF1α* modulators represent rational candidates. In fibrotic-prone cases, localized TGF-β/Notch or YAP/TAZ pathway modulation is proposed to inhibit fibroblast activation. These targeted agents are delivered via lung-optimized carriers such as aerosolized nanoparticles or engineered exosomes to enhance epithelial specificity while minimizing systemic exposure. This strategy aligns with evolving precision medicine paradigms but requires further validation in ARDS-specific contexts.

Therapeutic efficacy is dynamically monitored using bedside biosensors, such as electrochemical assays for BALF biomarkers and exhaled miRNA panels, enabling dose adjustments based on biological feedback. Patient-derived lung organoids and lung-on-chip platforms support preclinical drug sensitivity testing, while AI-driven decision systems integrate clinical and multi-omics data to guide regimen optimization. Collectively, this framework shifts ARDS management toward precision regenerative medicine through synchronized endotype targeting, adaptive delivery, and biosensor-guided monitoring.

## 7. Conclusions

ARDS is characterized by AEC dysfunction, which underlies barrier disruption, impaired fluid clearance, and dysregulated inflammatory signaling [19]. This pathophysiological cascade is driven by multiple mechanisms, including NF-κB activation, ROS-mediated oxidative stress, and epigenetic modifications. Recent applications of spatial transcriptomics and scRNA-seq reveal profound epithelial heterogeneity in ARDS. Transitional progenitor cells accumulate while AT2-to-AT1 differentiation is impaired, and distinct AEC subtypes actively remodel local immune microenvironments. These insights advance mechanistic understanding and highlight novel therapeutic targets.

Conventional therapies such as antioxidants, anti-inflammatory agents, and growth factors can provide transient protection of epithelial integrity and promote regeneration. However, inconsistent therapeutic efficacy and off-target toxicity frequently limit their clinical application [19,114]. Precision medicine strategies aim to overcome these limitations through biomarker-guided patient stratification, CRISPR-Cas9 genome editing, and nanoparticle-based drug delivery [181]. Single cell endotyping identifies individuals with impaired regenerative capacity. For such patients, combination therapy employing keratinocyte growth factor (KGF) and MSCs may enhance epithelial repair efficacy. LNP-encapsulated siRNA mediates localized anti-inflammatory effects within damaged alveolar areas.

The clinical translation of individualized strategies requires overcoming critical barriers. First, integrated analysis of multi-omics datasets is required to identify actionable therapeutic targets [182]. Second, interoperable analytical workflows enable cohesive collaboration integrating molecular biology, bioengineering, computational, and clinical practice [61,182]. Third, ensuring CRISPR-Cas9 safety requires high-fidelity nucleases and rigorous genomic screening to minimize off-target effects. Fourth, thorough preclinical and clinical assessment of nanocarriers under Good Manufacturing Practice (GMP) must evaluate biodistribution patterns, toxicity profiles, and cost-effective manufacturing scalability [182,183]. Finally, widespread adoption of spatial and single-cell technologies requires standardized protocols, AI-assisted tissue processing, and multicenter registries for longitudinal tracking of biomarkers, genomic features, and clinical outcomes.

A multilevel translational strategy integrates high-resolution spatial omics empowered by multiplexed in situ hybridization and AI modeling. This approach further combines advanced human-relevant models such as lung-on-chip systems, epithelial-immune co-cultures and patient-derived organoids. This integrated approach accelerates validation of nanoparticle biodistribution and gene-editing precision [184,185]. Concurrently, de-identified multicenter biomarker registries will guide adaptive clinical trial designs and real-world evidence synthesis [186,187]. By coupling targeted delivery with molecular mapping, this framework shifts ARDS management from symptomatic support toward reestablishing epithelial-immune homeostasis. Such targeted interventions may improve survival, functional recovery, and long-term quality of life.

## Figures and Tables

**Table 2:** Summary of Novel Epithelial-Targeted Therapeutic Strategies for ARDS.

Strategy	Target/Mechanism	Representative Agents/Approaches	Stage
Antioxidants	Neutralize ROS, reduce oxidative stress	N-acetylcysteine (NAC), Melatonin	Clinical trials (NAC), Preclinical (Melatonin)
Broad-Spectrum Anti-inflammatories	Inhibit NF-κB signaling, broadly reduce cytokine production	Glucocorticoids (e.g., Methylprednisolone)	Clinical use
Targeted Anti-inflammatories	Block specific cytokine receptors (e.g., IL-1R), inhibit pyroptosis	Anakinra (IL-1RA)	Clinical trials
Cell Therapy	Paracrine immunomodulation and pro-repair effects	Mesenchymal stem/stromal cells (MSCs)	Clinical trials
Gene Therapy & Editing	Therapy: Deliver protective genes. Editing: Correct dysfunctional genes.	AAV vectors (therapy), CRISPR-Cas9 (editing)	Preclinical
Exosomes	Deliver bioactive cargo (e.g., miRNAs) to modulate inflammation and repair	MSC-derived exosomes, miR-146a-5p loaded exosomes	Preclinical
Synthetic Nanodelivery	Targeted delivery of drugs/genes to lung cells; suppress inflammation	TLR4 siRNA-LNPs, Mannosylated miR-146a LNPs	Preclinical
Precision Medicine	Biomarker-guided patient stratification for targeted therapy	sRAGE, exosomal miRNAs, scRNA-seq-defined subsets	Exploratory/Research
Microbiome Modulation	Restore gut-lung axis, reduce systemic inflammation, enhance barrier	Probiotics, SCFAs/Butyrate, FMT	Preclinical

AAV, adeno-associated virus; ARDS, acute respiratory distress syndrome; CRISPR-Cas9, clustered regularly interspaced short palindromic repeats-associated protein 9; FMT, fecal microbiota transplantation; IL-1R, interleukin-1 receptor; LNPs, lipid nanoparticles; miRNAs, microRNAs; MSCs, mesenchymal stem cells; NAC, N-acetylcysteine; NF-κB, nuclear factor kappa-light-chain-enhancer of activated B cells; PLGA, poly (lactic-co-glycolic acid); ROS, reactive oxygen species; scRNA-seq, single-cell RNA sequencing; SCFAs, short-chain fatty acids; sRAGE, soluble receptor for advanced glycation end products; TLR4, toll-like receptor 4; TNF-α, tumor necrosis factor-alpha.

## Data Availability

No new data were created.

## Abbreviations

The following abbreviations are used in this manuscript:

ALI	acute lung injury
ARDS	acute respiratory distress syndrome
AEC	alveolar epithelial cell
PF	pulmonary fibrosis
AT1 cells	alveolar type I epithelial cells
AT2 cells	alveolar type II epithelial cells
ECM	extracellular matrix
TNF-α	tumor necrosis factor-α
IL-1β	interleukin-1β
MOMP	mitochondrial outer membrane permeabilization
FADD	Fas-associated death domain protein
GSDMD	gasdermin D
PAMPs	pathogen-associated molecular patterns
DAMPs	damage-associated molecular patterns
MLKL	mixed lineage kinase domain-like protein
ROS	reactive oxygen species
ENaC	epithelial sodium channels
*AQP5*	aquaporin 5
HMGB1	High Mobility Group Box 1
TLR4	Toll-like receptor 4
JNK	the c-Jun N-terminal kinase
MAPK	mitogen-activated protein kinase
JAK	Janus kinase
STAT3	Signal Transducer and Activator of Transcription 3
HATs	histone acetyltransferases
lncRNAs	Long non-coding RNAs
SDHA	succinate dehydrogenase A
CXCL1	Chemokine Ligand 1
LPS	lipopolysaccharide
BLM	bleomycin
scRNA-seq	single-cell RNA sequencing
VEGF	Vascular Endothelial Growth Factor
VE-cadherin	Vascular Endothelial Cadherin
GM-CSF	granulocyte-macrophage colony-stimulating factor
SCFAs	Short-chain fatty acids
TMAO	trimethylamine N-oxide
FMT	fecal microbiota transplantation
NAC	N-acetylcysteine
AAV	Adeno-associated virus
LNPs	Lipid nanoparticles
PLGA	poly(lactic-co-glycolic acid)
BALF	bronchoalveolar lavage fluid
DATP	damage-associated transient progenitor
TEER	transepithelial electrical resistance
